# Uric acid quantification *via* colorimetric detection utilizing silver oxide-modified activated carbon nanoparticles functionalized with ionic liquid

**DOI:** 10.1039/d4ra00659c

**Published:** 2024-02-27

**Authors:** Umar Nishan, Ateeq Ahmed, Nawshad Muhammad, Mohibullah Shah, Muhammad Asad, Naeem Khan, Farman Ullah, Riaz Ullah, Essam A. Ali, Haq Nawaz, Amir Badshah

**Affiliations:** a Department of Chemistry, Kohat University of Science and Technology Kohat 26000 KP Pakistan umarnishan85@gmail.com amirqau@yahoo.com; b Department of Dental Materials, Institute of Basic Medical Sciences Khyber Medical University Peshawar KPK Pakistan; c Department of Biochemistry, Bahauddin Zakariya University Multan 66000 Pakistan; d Department of Pharmacy, Kohat University of Science and Technology Kohat 26000 KP Pakistan; e Department of Pharmacognosy, College of Pharmacy, King Saud University Riyadh Saudi Arabia; f Department of Pharmaceutical Chemistry, College of Pharmacy, King Saud University Riyadh Saudi Arabia; g School of Chemistry and Chemical Engineering, Huaiyin Normal University 223300 Huaiyin Jiangsu China

## Abstract

Uric acid (UA) is a significant indicator of human health because it is linked to several diseases, including renal failure, kidney stones, arthritis, and gout. Uric acid buildup in the joints is the source of chronic and painful diseases. When UA is present in large quantities, it causes tissue injury in the joints that are afflicted. In this research, silver oxide-doped activated carbon nanoparticles were synthesized and then functionalized with an ionic liquid. The synthesized nanomaterial assembly was employed as a colorimetric sensing platform for uric acid. Activated carbon offers a large internal surface area that acts as a good carrier for catalytic reactions. A salt-melting approach was used to synthesize the silver oxide-doped activated carbon nanocomposite. The synthesis was confirmed through various techniques, such as UV-vis spectrophotometer, FTIR, XRD, SEM, and EDX. The colorimetric change from blue-green to colorless was observed with the naked eye and confirmed by UV-vis spectroscopy. To obtain the best colorimetric change, several parameters, such as pH, capped NP loading, TMB concentration, hydrogen peroxide concentration, and time, were optimized. The optimized experimental conditions for the proposed sensor were pH 4 with 35 μL of NPs, a 40 mM TMB concentration, and a 4 minutes incubation time. The sensor linear range is 0.001–0.36 μM, with an *R*^2^ value of 0.999. The suggested sensor limits of detection and quantification are 0.207 and 0.69 nM, respectively. Potential interferers, such as ethanol, methanol, urea, Ca^2+^, K^+^, and dopamine, did not affect the detection of uric acid.

## Abstract 2

Uric acid (UA) is a significant indicator of human health because it is linked to several diseases, including renal failure, kidney stones, arthritis, and gout.

## Graphical abstract

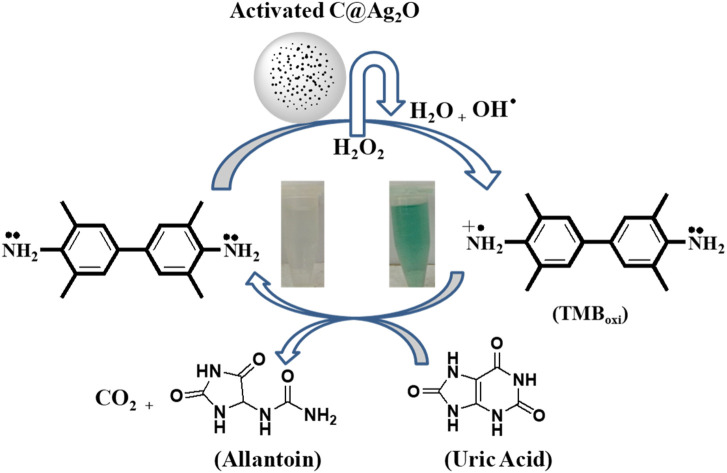

## 1 Introduction

The final product of purine derivatives in human metabolism is uric acid (UA). It is eliminated by the intestines and kidneys.^1^ Nutrition, age, drug use, and overall health all affect the amount of UA in the human body at varying concentrations. The range of UA in human serum is 120–460 μM.^2^ Serious conditions such as Lesch Nyhan syndrome, renal disorders, and gout can be brought on by abnormally high levels of UA.^3^ High UA levels, frequently referred to as hyperuricemia, are linked to hypertension, cardiovascular and cerebrovascular disorders, and metabolic syndrome.^4^ Thus, it is very crucial to develop a sensitive, accurate, and rapid platform for the detection of UA.

A number of techniques have been used for the determination of UA, such as enzymatic methods,^5^ chemiluminescence,^6^ liquid chromatography,^7^ and electrochemiluminescence.^8^ However, these methods have limited applications because of the high cost and complexity of the procedures, thereby restricting their application in labs with limited resources. In comparison to the previously described methods, the colorimetric biosensor method for UA detection is extremely quick, easy to use, inexpensive, highly selective, and sensitive. Since the discovery of Fe_3_O_4_ nanoparticles as enzyme mimics,^9^ an increasing number of scientists have shown that nanozymes possess peroxidase-like activity. They effectively overcome the shortcomings of natural enzymes, such as their high cost, low shelf life, difficult handling, and instability.^10^

Activated carbons (ACs) have recently attracted a lot of attention due to their favorable physico- and electro-chemical characteristics. These include, accessibility, surface functionality, good electrical conductivity, thermal stability, low toxicity, uniform porosity, and high surface area.^11^ AC is a material that meets all of the requirements for usage as a catalyst support. In comparsion to conventional carriers like alumina or silica, ACs offer a larger internal surface area for a higher reaction rate and a lower cost per cubic meter. In the hydrothermal process, the addition of a tiny amount of metal ions to activated carbon led to the creation of carbon nanoparticles and gave them strong peroxidase-like activity and use in various aspects.^12^ The ability of iron nanoparticles was increased when they were modified with activated carbon compared to pure activated carbon.^13^

The capability of nanoparticles to facilitate quick electron transport between the electrode and the enzyme's active site is one of its special qualities.^14^ Various types of nanoparticles, including gold (AuNPs),^15^ zinc-oxide (ZnO-NPs),^16^ and iron-oxide (Fe_3_O_4_-NPs),^17^ have been used for enzyme immobilization to improve the stability and sensitivity of the activated carbon based biosensors. In addition, modified activated carbons are utilized in a variety of processes, including gas and air purification, water treatment, catalysis, metal extraction, energy storage, and bio and environmental applications. A large variety of chemical substances and bacterial nutrients can be effectively absorbed by AC.^18^

Ionic liquids are of great interest due to their unique properties, including low volatility, relatively strong ionic conductivity, chemical and thermal stability, and the capacity to dissolve a variety of compounds by appropriately altering the anions and cations.^19^ They can be used as a capping agent to improve the stability, electrical conductivity and deagglomeration of nanomaterials.^20^

In the current work, we have fabricated silver oxide-doped activated carbon using a salt melting method. The method as opposed to solvent based methods is free from the temperature limitation and can operate on temperatures as high as 1000 °C. Furthermore, the synthesized platform was functionalized with 1-H-3 methyl imidazolium acetate ionic liquid. The novelty of the current work lies in the use of silver oxide-doped activated carbon functionalized with ionic liquid for the colorimetric sensing of uric acid. The reported method is highly sensitive, selective, rapid, and simple. The chromogenic substrate 3,3′,5,5′-tetramethylbenzidine (TMB) was used with the support of H_2_O_2_. To obtain the best colorimetric response, various parameters, such as TMB concentration, ionic liquid-coated NPs, incubation time, and pH were optimized. The sensitivity and selectivity of the proposed sensor were also analyzed using the optimum conditions. The optimized platform was used successfully to detect UA in human blood serum samples with excellent sensitivity and selectivity.

## 2 Materials and methods

### 2.1 Chemicals and reagents

Sigma Aldrich provided 3,3′,5,5′-tetramethylbenzidine (TMB), sodium hydroxide (NaOH), hydrochloric acid (HCl), silver nitrate (AgNO_3_), and hydrogen peroxide (H_2_O_2_). BioWorld provided PBS in a range of pH levels. All of these compounds were in their pure form and were used without further purification. The solutions were made with deionized water from an Elga Purelab Ultra water deionizer, and the tests were carried out with high-quality glassware. Human serum samples were collected from a local lab (Al-Habib clinical laboratory) in Kohat and diluted to reduce the serum complexity and also to take advantage of the sensitive nature of the proposed sensor. This work was approved by the ethical committee of the Kohat University of Science and Technology, Kohat KP Pakistan *via* KUST/Ethical Committee/22-12.

### 2.2 Instrumentation

The FTIR spectrometer used was from Agilent Technologies in Danbury, Conn. The FTIR spectra of the materials were obtained using a range of 4000–500 cm^−1^. Scanning electron microscopy (SEM) was used to examine the morphology of silver oxide-doped activated carbon nanoparticles. X-ray powder diffraction (XRD; PAN analytical, X'pert Powder) was used to analyze the size and phase of the synthesized silver oxide-doped activated carbon nanoparticles. On a UV-vis spectrophotometer, the UV-vis spectra of both the silver oxide-doped activated carbon nanoparticles and the experimental materials were recorded (Shimadzu, UV, 1800, Japan).

### 2.3 Synthesis of silver oxide doped activated carbon

An innovative and inexpensive salt melting method was employed to synthesize silver oxide-doped activated carbon nanoparticles by utilizing a modified version of the method described elsewhere.^21,22^ Briefly, 5 g of silver nitrate and 10 g of activated carbon were mixed using a mortar and pestle. The combined samples were then placed in a muffle furnace in a crucible with a volume of 30 mL. The mixed sample was stored for four hours after being heated at a rate of 10 °C per minute from room temperature to 800 °C. The muffle furnace's interior was used to cool the activated sample to room temperature. After that, the synthesized NPs were processed for characterization.

### 2.4 Characterization

The prepared nanoparticles were characterized using different techniques. These include Fourier transform infrared spectroscopy (FTIR), scanning electron microscopy (SEM), X-ray diffraction (XRD), thermogravimetric analysis (TGA), EDX, and UV-vis spectroscopy.

### 2.5 Synthesis of ionic liquid

The preparation of 1-H-3 methylimidazolium acetate ionic liquid was done using the modified protocol previously reported by our group.^23,24^

### 2.6 Capping of silver oxide doped activated carbon NPs with ionic liquid

The synthesized platform (silver oxide doped AC) was functionalized by using 1-H-3-methyl imidazolium acetate ionic liquid (IL) as capping and deagglomeration agent. 1 mL of the ionic liquid was mixed with the 6 mg of the synthesized silver-oxide doped activated carbon. Using a pestle and mortar, the mixture was vigorously ground for thirty minutes. The ionic liquid-functionalized Ag_2_O nanoparticles were stored for further analysis. The functionalization of Ag with ionic liquid was also reported by Asad *et al.*^25^

### 2.7 Colorimetric detection of uric acid

The peroxidase-like activity of silver-doped activated carbon NPs was evaluated through colorimetric detection. In this method, 3,3′,5,5′-tetramethylbenzidine (TMB) is expected to be oxidized by H_2_O_2_ from a colorless to a blue-green product. The procedure will be carried out as follows: 50 μL of stock solution of silver-doped activated carbon NPs and 200 μL of TMB (2 mM) and 0.2 mM acetate buffer solution were mixed, followed by the addition of 200 μL of hydrogen peroxide. Subsequently, 150 μL of UA (0.36 μM) was added to the reaction solution and incubated. The expected colorimetric change was observed with the naked eye and confirmed with a UV-vis spectrophotometer.

## 3 Results and discussion

### 3.1 FTIR analysis

**Fig. 1:**
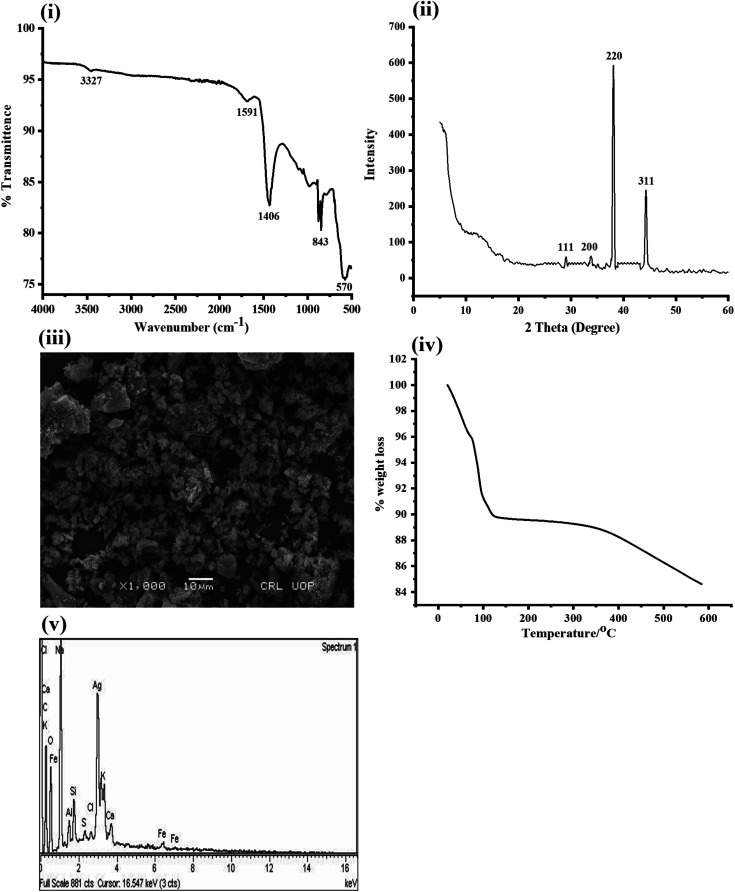
(i) FTIR spectrum of Ag_2_O-doped activated carbon showing the presence Ag–O bond. (ii) XRD analysis of the synthesized Ag_2_O-doped activated carbon indicating the orthorhombic-phase. (iii) SEM image of the synthesized Ag_2_O-doped activated carbon NPs. (iv) TGA analysis of the prepared Ag_2_O-doped activated carbon NPs. (v) EDX analysis of the prepared Ag_2_O nanoparticles.

Fourier transform infrared spectroscopy was used for the determination of functional groups. Fig. 1(i) demonstrates the FTIR spectrum of the prepared silver oxide-doped activated carbon nanoparticles. The peak present at 3327 cm^−1^ reflect the presence of OH in our synthesized platform. The OH band at 3327 cm^−1^ usually comes from moisture that is present in the air. The peaks around 1591 cm^−1^ indicate the presence of C=C in conjugation with other functionalities. The other bands at 1406 cm^−1^ reflect the presence of alkyl bending functionalities. The band around 843 cm^−1^ is due to the presence of a C–Cl group present in the activated carbon. The final band at 570 cm^−1^ can be attributed to the presence of Ag–O in the platform.^26^

#### 3.1.1 XRD analysis

Fig. 1(ii) shows the X-ray diffraction pattern of Ag_2_O-doped activated carbon NPs. The XRD pattern of the synthesized Ag_2_O-doped activated carbon NPs shows two diffraction peaks centered at 2*θ* = 29, 33, 38, and 44 with miller indices of 111, 200, 220, and 311. The production of Ag_2_O-doped activated carbon NPs with an orthorhombic phase is confirmed by the diffraction peaks in the XRD pattern of synthesized Ag_2_O-doped activated carbon NPs (JCPDS No. 047-0561).^27^ The average crystal sizes of orthorhombic-phase Ag_2_O-doped activated carbon NPs were estimated to be about 17 nm.

#### 3.1.2 SEM analysis

Fig. 1(iii) shows the SEM image of the synthesized Ag_2_O-doped activated carbon NPs. The image shows that the Ag_2_O nanoparticles distributed homogenously on the surface of the activated carbon. Here the activated carbon plays important role in keeping the Ag_2_O nanoparticles in non-agglomerated form, As a result the activity of our synthesized platform is very high, which act as nanoenzyme.^28^

#### 3.1.3 TGA analysis

The thermal degradation behavior (TGA curve) of the silver oxide-doped activated carbon NPs is shown in Fig. 1(iv). It is observed from the TGA curve that weight loss of the NPs occurred throughout the temperature range of 30 to 600 °C. The first weight loss can be attributed to the loss of moisture. The second weight loss can attributed to the organic moieties present in the activated carbon.^29^

#### 3.1.4 EDX spectrum

The prepared silver oxide-doped activated carbon nanoparticles were utilized for the study of the energy-dispersive X-ray spectrum. Ag peaks can be clearly seen in Fig. 1(v). In the EDX spectrum of silver oxide-doped activated carbon nanoparticles, the peaks for the elements O and C could be seen. Using an EDX spectrum, the chemical compositions of the Ag_2_O/AC NPs were examined. The composites contained Ag 3.79% in atomic percentage, 32.66% oxygen, and 51.64% carbon.^3^ Various other elements, such as Na, Al, Si, *etc.*, were also detected in minor quantities (Table 1), which are typically observed in activated carbon depending on the source of its origin and the reagents used for its preparation.

**Table 1:** EDX analysis showing the elemental composition of the synthesized Ag_2_O-doped AC NPs

Element	Weight%	Atomic%
C	33.56	51.64
O	28.42	32.66
Na	8.01	6.80
Al	1.24	0.90
Si	1.96	1.36
S	0.44	0.27
Cl	0.24	0.13
K	2.68	1.34
Ca	1.47	0.72
Fe	1.11	0.39
Ag	20.87	3.79
Totals	100.0	100.0

### 3.2 Colorimetric detection of uric acid

**Fig. 2:**
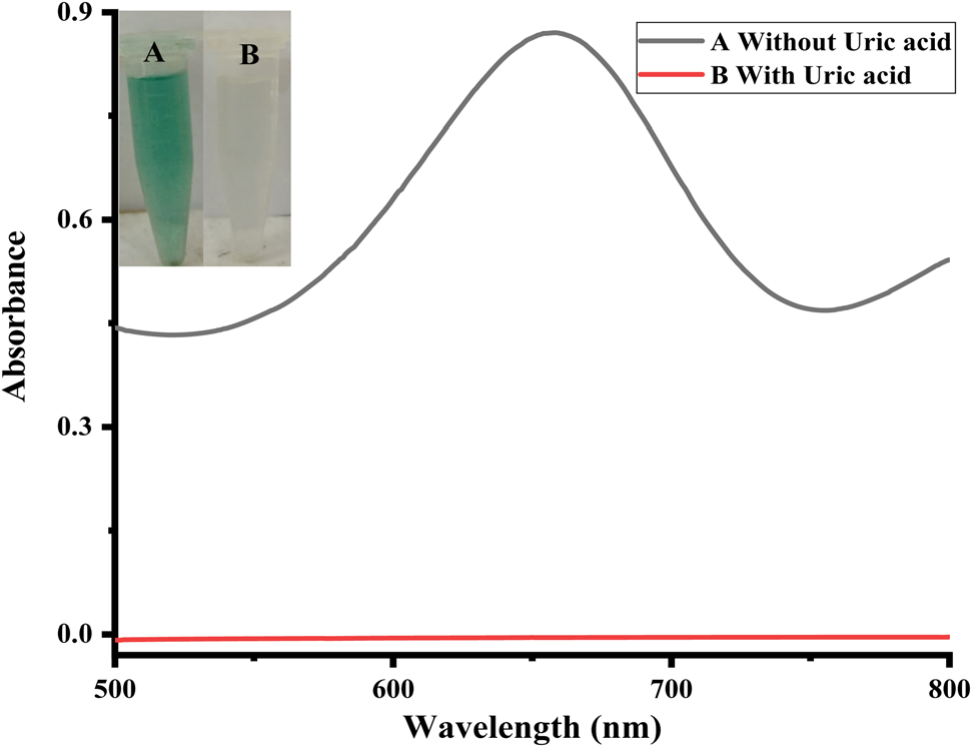
Colorimetric detection of uric acid [conditions: capping 35 μL, PBS 550 μL, TMB 200 μL (40 mM), H_2_O_2_ 90 μL (25 mM), uric acid 150 μL (0.36 μM)].

For the detection of uric acid, a simple and selective colorimetric technique based on Ag_2_O-doped activated carbon NPs was used. Fig. 2 depicts the optical sensing and UV-vis absorption spectra for the reaction mixture. The mimic enzyme with the assistance of hydrogen peroxide converts the colorless TMB solution to blue-green color (TMB_oxi_). The addition of uric acid to the reaction mixture resulted in the reduction of oxidized TMB to reduced form. This transformation was observed with the naked eye and the color of TMB solution diminished. The colorimetric change was confirmed through a UV-vis spectrophotometer as shown in the Figure.

### 3.3 Ionic liquid-capped Ag_2_O/activated carbon NPs based sensing of UA

**Scheme 1:**
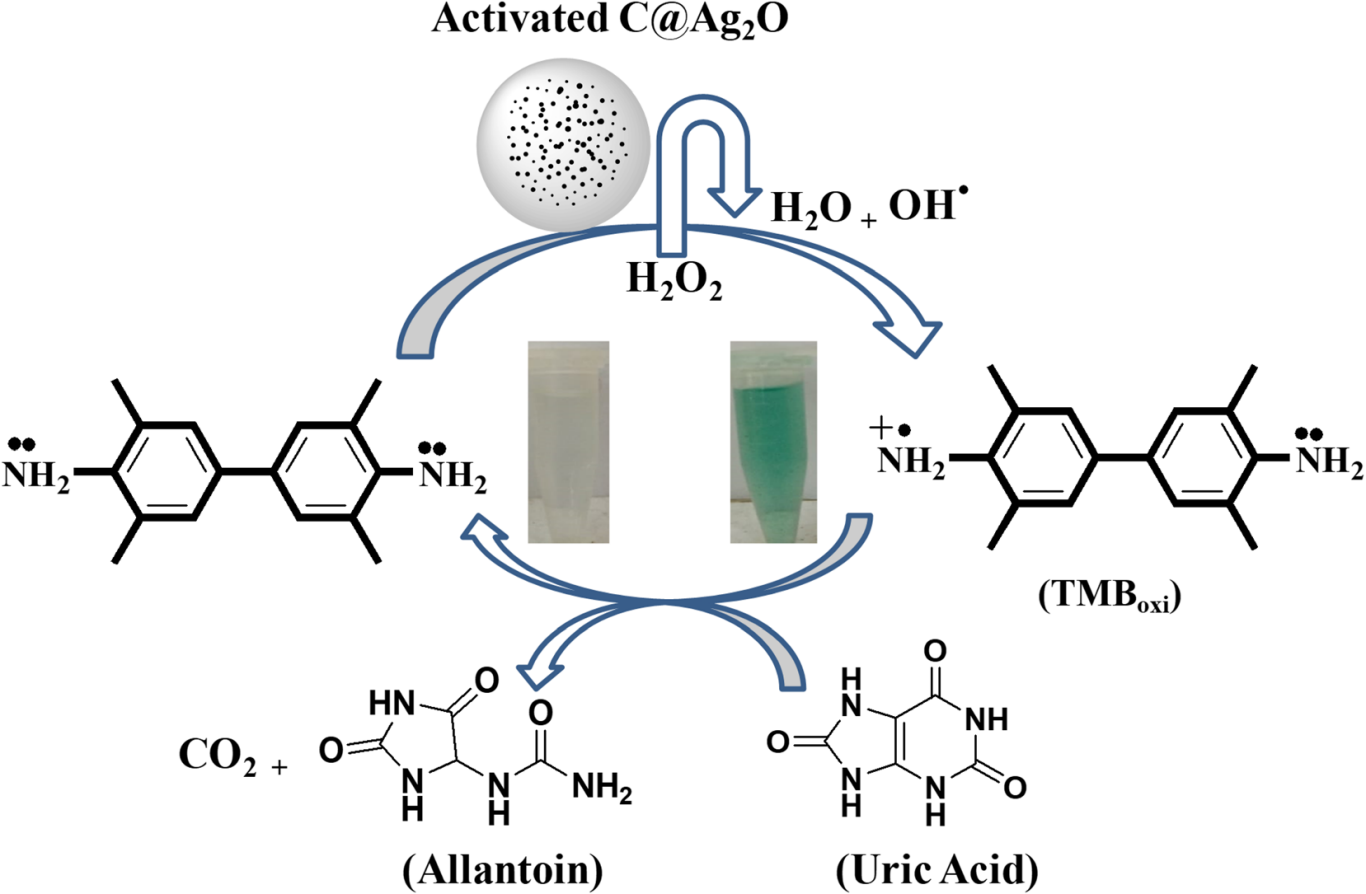
Ionic liquid capped Ag_2_O/activated carbon NPs as a sensing platform for uric acid.

In this work, we propose a simple, sensitive, and selective colorimetric approach for uric acid detection based on Ag_2_O-doped activated carbon NPs. Ag_2_O/AC NPs have intrinsic oxidase-like activity, and in the presence of hydrogen peroxide, TMB can be oxidized from colorless to blue-green oxidized TMB, generating an absorption peak at 652 nm as shown in Scheme 1. During the course of the reaction, the mimic enzyme catalyzes the production of OH free radicals from H_2_O_2_. The generated free radicals oxidize the TMB, and hence a blue-green color is produced, indicating the presence of an oxidized form of TMB. The reduction of oxidized TMB after the addition of uric acid in the presence of Ag_2_O/AC NPs decreased proportionally until it became completely transparent. During the reduction of TMB to its colorless form, the uric acid acts as a reducing agent. It reduces the oxidized TMB to its original, colorless form and itself oxidizes to allantoin along with the production of CO_2_, as shown in Scheme 1.

### 3.4 Optimization of parameters

#### 3.4.1 Capped NPs amount optimization

**Fig. 3:**
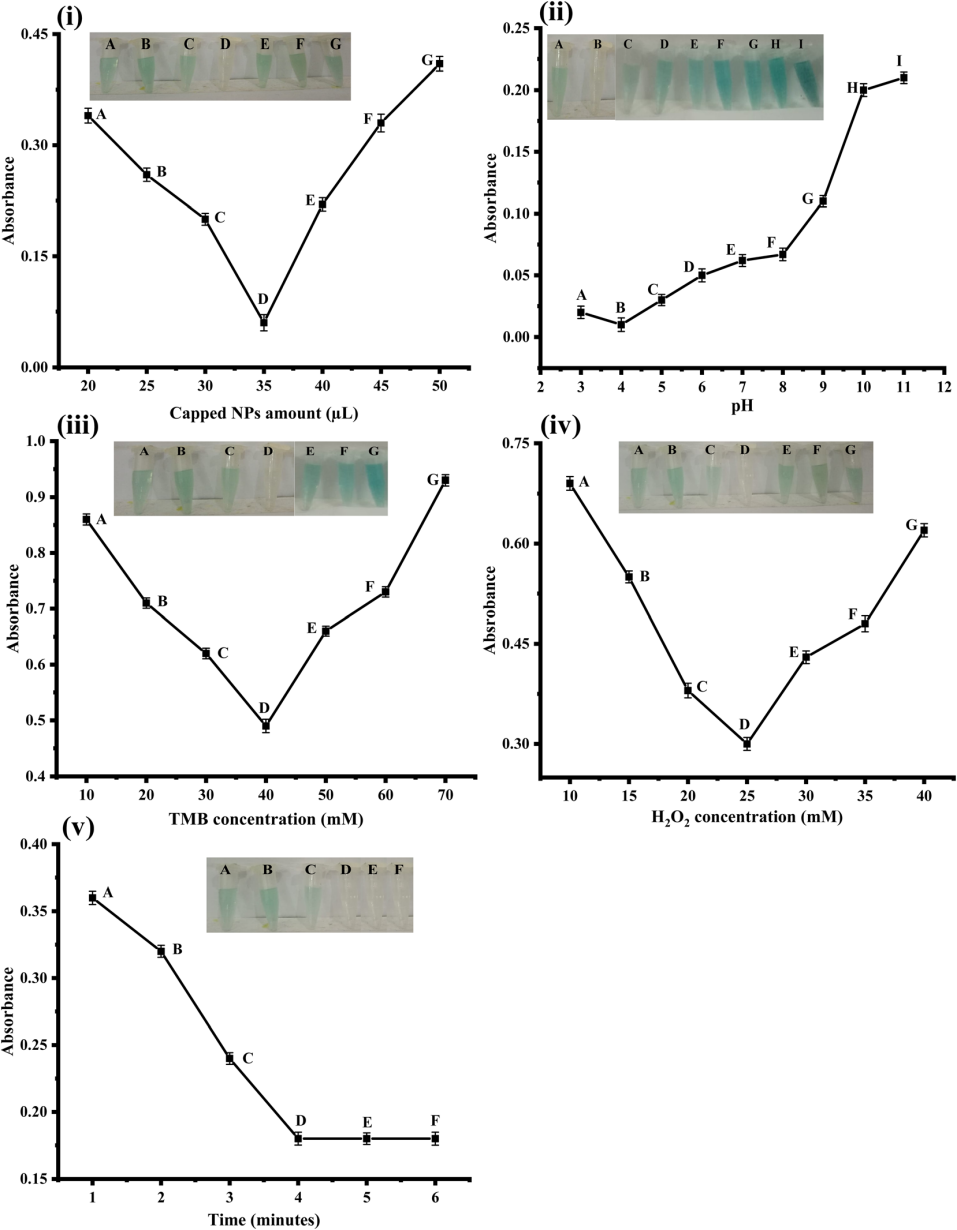
(i) Optimization of capped amount [conditions: capping 35 μL, PBS 550 μL, TMB 200 μL (40 mM), H_2_O_2_ 90 μL (25 mM), uric acid 150 μL (0.36 μM)]. (ii) pH optimization of the proposed sensor [conditions: capping 35 μL, PBS 550 μL, TMB 200 μL (40 mM), H_2_O_2_ 90 μL (25 mM), Uric acid 150 μL (0.36 μM)]. (iii) TMB optimization of the proposed sensor [conditions: capping 35 μL, PBS 550 μL, TMB 200 μL (40 mM), H_2_O_2_ 90 μL (25 mM), uric acid 150 μL (0.36 μM)]. (iv) Hydrogen peroxide optimization of the proposed sensor [conditions: capping 35 μL, PBS 550 μL, TMB 200 μL (40 mM), H_2_O_2_ 90 μL (25 mM), uric acid 150 μL (0.36 μM)]. (v) Time optimization of the proposed sensor [conditions: capping 35 μL, PBS 550 μL, TMB 200 μL (40 mM), H_2_O_2_ 90 μL (25 mM), uric acid 150 μL (0.36 μM)].

The amount of capped nanoparticles was optimized, as shown in Fig. 3(i). Different amounts of NPs were taken in an Eppendorf tube, and the best colorimetric response was obtained at 35 μL of NPs, as shown in Fig. 3(i). When the amount of NPs was either increased or decreased from 35 mg, the colorimetric response was not as good as in the case of 35 mg. So a 35 μL amount was considered the optimum amount and was used in further experiments. Previously, Nishan *et al.* reported that 40 μL of IL-capped NiNPs was optimal for the detection of uric acid.^30^

#### 3.4.2 pH optimization

The pH of the solution is critical in the biosensor system. It changes the biosensor's efficiency by either increasing or decreasing it. As shown in Fig. 3(ii), the influence of pH on the sensor was investigated using PBS buffer solution at various pH levels ranging from 3 to 11. PBS buffer solution was used to optimize the pH, while hydrochloric acid and sodium hydroxide solutions were used to change the pH. At pH 4, there was a good response. A modest colorimetric change was found at pH 3 and pH 5. There was no colorimetric change at higher pH, ranging from 6 to 11, and at lower pH 3, implying that pH 4 provided the optimum colorimetric response. As a result, pH 4 was chosen as the best pH for further experiments. pH less than 4 was used in the literature because catalytic activity in acidic solutions was substantially higher than in neutral and basic solutions.

#### 3.4.3 TMB optimization

The TMB concentration was also optimized to achieve the best colorimetric change. TMB solutions of various concentrations were prepared, ranging from 10 mM to 70 mM. The sensor responded best at a 40 mM TMB concentration, and when uric acid was added, the color became light and transparent. The colorimetric reaction was not as good at lower concentrations or higher concentrations as 40 mM. For the rest of the experiments, a concentration of 40 mM TMB was chosen as the optimal concentration, as shown in Fig. 3(iii).

#### 3.4.4 Hydrogen peroxide optimization

The hydrogen peroxide concentration was also optimized to obtain the best colorimetric response, as shown in the figure. Different concentrations of hydrogen peroxide were formed, ranging from 10 mM to 40 mM, and the best colorimetric response was obtained at a 25 mM concentration. At concentrations higher or lower than 25 mM, no significant colorimetric change was observed, so the 25 mM concentration was chosen as the optimum concentration, and further experiments were performed using this optimum concentration, as shown in Fig. 3(iv).

#### 3.4.5 Time optimization

The impact of time on the sensor was also investigated at intervals ranging from 1 to 6 minutes. The best response was obtained after 4 minutes of incubation because all of the capped NPs were consumed in the reaction, no additional changes were detected after 4 minutes, and the color became fully transparent. As a result, for the remaining experiments, 4 minutes was chosen as the optimal time, as shown in Fig. 3(v).

#### 3.4.6 Colorimetric and spectrophotometric detection of uric acid

**Fig. 4:**
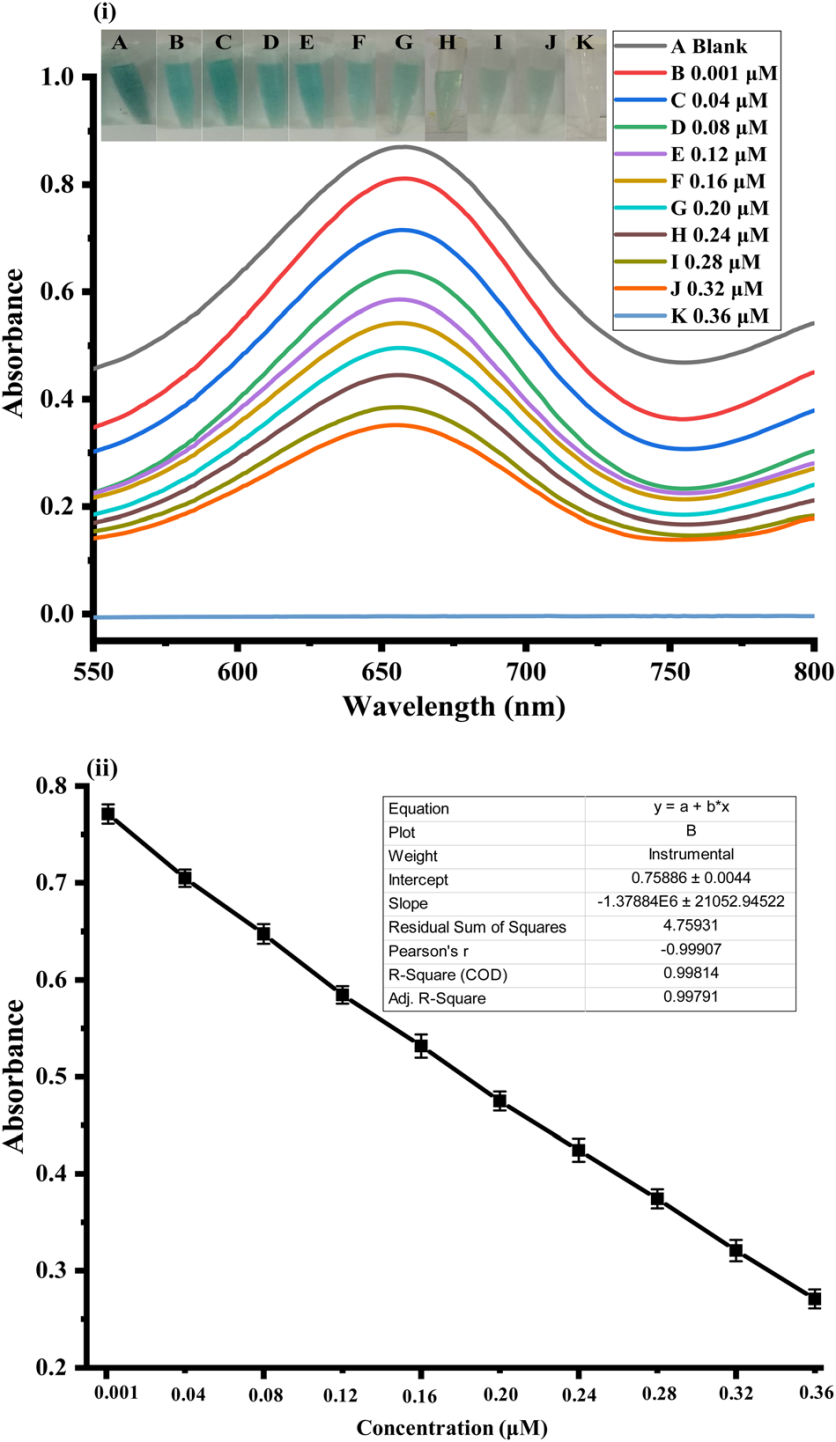
(i) Typical UV-vis spectra of the proposed assay at various UA concentrations (0.001–0.36 μM), Ag_2_O/AC, H_2_O_2_, and TMB. (ii) Relationship between the UA concentration and the absorption spectra peak at 652 nm. The error bars show the standard deviations of three different measurements.

To detect different concentrations of uric acid, a simple colorimetric test was used directly using the Ag_2_O-doped activated carbon NPs under optimum experimental conditions. For the quantitative assessment of uric acid, a sensitive and selective colorimetric technique based on the relationship between uric acid concentration and absorbance intensity at 652 nm was used. Various uric acid concentrations were used to test the sensitivity of the sensor designed for uric acid detection. Fig. 4(i) and (ii) show the response of colorimetric biosensors to various uric acid concentrations. The sensor response and peak intensity were high at lower uric acid concentrations, but they dropped linearly as the uric acid concentration increased. With an *R*^2^ value of 0.999, this method was able to detect uric acid with a linear range of 0.001–0.36 μM. The limits of quantification (LOQ) and detection (LOD) were determined to be 0.69 and 0.207 nM, respectively. In comparison to other previously reported detection methods, the proposed colorimetric method had the advantages of a low detection limit, low cost, and direct eye observation. This shows that our sensor can work at both high and low concentrations. Based on the linear range and limit of detection, the following Table 2 compares previously reported colorimetric methods with the present study for uric acid detection.

**Table 2:** Comparison of some of the colorimetric biosensors for uric acid with recently reported studies

S. No	Materials used	Method	Limit of detection (μM)	Linear range (μM)	References
1	Ag nanoprisms	Colorimetric	0.7	1 to 40	31
2	MIL-53(Fe)	Colorimetric	1.3	4.5 to 60	32
3	MoS_2_ nanoflakes	Colorimetric	0.3	0.5–100	33
4	Chitosan stabilized Au NPs	Colorimetric	0.04	0.1 to 30	34
5	AuNPs-GO	Colorimetric	0.06	1 to 40	35
6	CuS NPs	Colorimetric	1	1–100	36
7	PtNPs	Colorimetric	4.2	1–8000	37
8	μPADs modified with chitosan	Colorimetric	37	130–380	38
9	Fe_3_O_4_@MnO_2_	Colorimetric	0.27	1–70	39
10	IL-Ag_2_O/activated carbon NPs	Colorimetric	0.00021	0.001–0.36	This work

### 3.5 Selectivity study of the proposed sensor

**Fig. 5:**
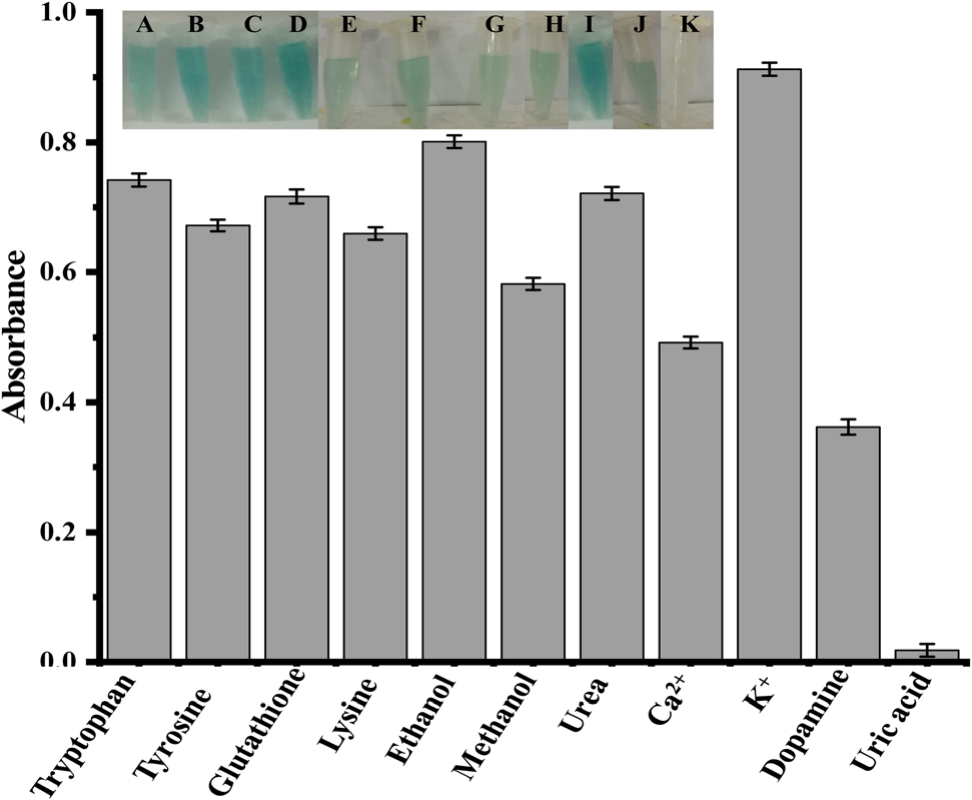
Selectivity study of the proposed sensor at optimized conditions [conditions: capping 35 μL, PBS 550 μL, TMB 200 μL (40 mM), H_2_O_2_ 90 μL (25 mM), tryptophan, tyrosine, glutathione, lysine, ethanol, methanol, urea, Ca^2+^, K^+^, and dopamine 150 μL (0.36 μM)].

Fig. 5 shows that the selectivity of the proposed sensor was tested with potential interfering species such as tryptophan, tyrosine, glutathione, lysine, ethanol, methanol, urea, Ca^2+^, K^+^, and dopamine. Tryptophan, tyrosine, glutathione, lysine, ethanol, methanol, urea, Ca^2+^, K^+^, and dopamine have relatively high absorption values when compared to uric acid. The absorption value at 652 nm decreases when uric acid is added, as indicated in Fig. 5. As shown in Fig. 5, there was no significant decrease in absorbance when other interfering species were added. So it is concluded that, in the presence of other interfering substances, our proposed sensor can detect uric acid in the presence of other potential interfering substances.

### 3.6 Applications of the proposed sensor

**Fig. 6:**
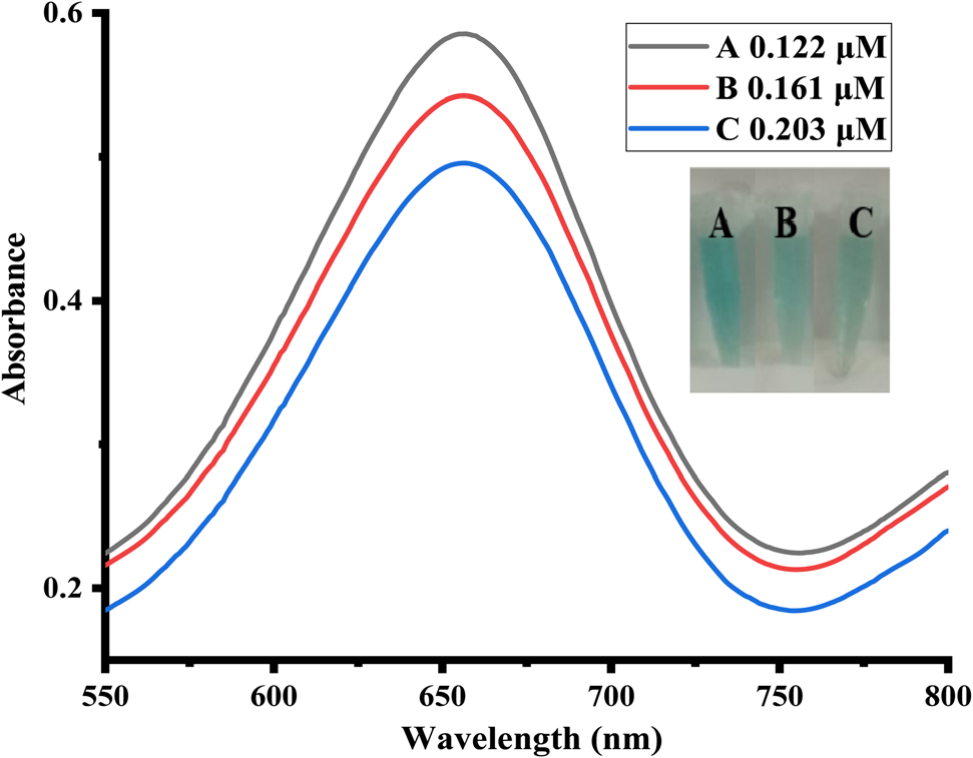
UV-vis spectra of uric acid detection in human blood serum samples (*n* = 3) by the dilution of the samples in line with the linear range of the fabricated sensor.

The human serum samples that were collected from the local lab were strategically diluted to bring their uric acid concentration within the linear range of the fabricated sensor. Briefly, using the previously drawn calibration plots and varying uric acid concentrations under the same experimental conditions. The concentration of uric acid in human blood serum samples was determined as shown in Fig. 6. Through this procedure, the values of uric acid in diluted serum samples were calculated to be 0.122, 0.161, and 0.203 μM.

## 4 Conclusion

In the present work, silver oxide-doped activated carbon nanoparticles were synthesized successfully by simple salt-melting. The functionalization of the synthesized material was done with an ionic liquid to achieve deagglomeration and better conductivity. The platform was successfully employed for the colorimetric detection of uric acid with exceptional sensitivity and selectivity. Comprehensive optimization experiments were performed to get the optimized conditions for the proposed sensor. The proposed sensor was applied to human serum samples for the successful detection of uric acid. The synthesized platform demonstrated intrinsic peroxidase-like activity. The utilization of silver oxide-doped activated carbon nanoparticles as peroxidase mimics for UA determination is thus considered a potential technique for bioassays and medical diagnostics.

## Conflicts of interest

The authors declare that there is no conflict of interests.

## Supplementary Material
